# Ketone Ester Supplementation Improves Some Aspects of Cognitive Function during a Simulated Soccer Match after Induced Mental Fatigue

**DOI:** 10.3390/nu14204376

**Published:** 2022-10-19

**Authors:** Manuel D. Quinones, Peter W. R. Lemon

**Affiliations:** Exercise Nutrition Research Laboratory, School of Kinesiology, The University of Western Ontario, London, ON N6A 3K7, Canada

**Keywords:** exogenous ketones, ketosis, soccer, intermittent exercise, cognition

## Abstract

Ketone supplementation has been proposed to enhance cognition during exercise. To assess whether any benefits are due to reduced cognitive fatigue during the latter portions of typical sport game action, we induced cognitive fatigue, provided a ketone monoester supplement (KME) vs. a non-caloric placebo (PLAC), and assessed cognitive performance during a simulated soccer match (SSM). In a double-blind, balanced, crossover design, nine recreationally active men (174.3 ± 4.2 cm, 76.6 ± 7.4 kg, 30 ± 3 y, 14.2 ± 5.5 % body fat, $\dot{V}$O_2_ max = 55 ± 5 mL·kg BM^−1^·min^−1^; mean ± SD) completed a 45-min SSM (3 blocks of intermittent, variable intensity exercise) consuming either KME (25 g) or PLAC, after a 40-min mental fatiguing task. Cognitive function (Stroop and Choice Reaction Task [CRT]) and blood metabolites were measured throughout the match. KME reduced concentrations of both blood glucose (block 2: 4.6 vs. 5.2 mM, *p* = 0.02; block 3: 4.7 vs. 5.3 mM, *p* = 0.01) and blood lactate (block 1: 4.7 vs. 5.4 mM, *p* = 0.05; block 2: 4.9 vs. 5.9 mM, *p* = 0.01) during the SSM vs. PLAC, perhaps indicating a CHO sparing effect. Both treatments resulted in impaired CRT performance during the SSM relative to baseline, but KME displayed a reduced (*p* < 0.05) performance decrease compared to PLAC (1.3 vs. 3.4% reduction in correct answers, *p* = 0.02). No other differences in cognitive function were seen. These data suggest that KME supplementation attenuated decrements in CRT during repeated, high intensity, intermittent exercise. More study is warranted to assess fully the potential cognitive/physical benefits of KME for athletes.

## 1. Introduction

In most team sports such as soccer, ice hockey, basketball, etc a significant cognitive and physical demand is imposed as athletes are required to make numerous, critical, split-second decisions while exercising at very high rates [1]. Often, fatigue occurs towards the end of a match and is associated with reduced glycogen stores [2,3]. During this time, athletes may experience physical fatigue evidenced by a decrement in work output [4,5], as well as increased mental fatigue exhibited as poor decision making and/or deterioration of skills [6,7,8]. This is important as the abovementioned factors are critical determinants of game play. In light of this, nutrient supplementation strategies to avoid or delay the onset of fatigue are of considerable interest to many.

Ketone bodies (KB) are lipid-derived metabolites produced in the liver during periods of low CHO availability such as starvation, prolonged exercise, uncontrolled diabetes, or dietary manipulations [9,10]. Aside from glucose and lactate, KB are the only energy substrate that can cross the blood–brain barrier doing so via monocarboxylate-mediated transport [11]. Consequently, KB are an alternative brain fuel which could enhance cognitive performance by reducing glucose reliance [11,12]. In addition, KB can increase the expression of the protein Brain Derived Neurotropic Factor (BDNF), an important molecule for brain plasticity/regulation of cognitive function [13,14]. Recently, ketone supplements have emerged as an alternative to a ketogenic diet to induce hyperketonemia acutely [15,16] and are available commercially in two forms, ketone salts (KS) and ketone esters (KE). These supplements have been shown to help spare CHO [15] and to provide an alternative fuel for the brain [17]. However, only a few studies have evaluated the efficacy of ketone supplements on cognitive function during exercise with some studies showing positive effects [18,19] while others found no benefits [20,21,22] when compared to CHO or a non-caloric placebo. This discrepancy in results has been attributed primarily to the use of KE [18,21] vs. KS [19,20,22] because the former can induce greater circulating KB [16], although some other methodological differences might also be important [23]. For example, most studies measured cognition before and after exercise as opposed to during exercise. The latter would have greater ecological validity as cognitive changes could occur rapidly in recovery from exercise. Further, it has been shown that cognitive function displays greater impairments when the tests and physical exercise are performed concurrently [24], possibly due to a dual-task interference. To our knowledge, only one ketone supplementation study has measured cognitive function during exercise. In this study, KS were given, which only induced slight to modest increases in circulating KB during steady state exercise [22]. The authors found no changes in cognition when compared to a non-caloric placebo. Whether the effects of exogenous KE are similar during high intensity, intermittent exercise, like that performed in many sports, is unknown. Therefore, the purpose of the present study was to assess whether acute ketone monoester (KME) supplementation can enhance/maintain cognitive performance during a 45-min simulated soccer match (SSM) after induced mental fatigue. We hypothesized that KME supplementation would enhance/maintain cognitive function during the SSM.

## 2. Materials and Methods

### 2.1. Participants

Nine healthy, male soccer players were recruited to participate in the study (height = 174.3 ± 4.2 cm, body mass = 76.6 ± 7.4 kg, age = 30 ± 3 y, body fat = 14.2 ± 5.5 %, and $\dot{V}$O_2_ max = 55 ± 5 mL· kg BM^−1^·min^−1^). All were experienced, recreational soccer players from a range of outfield playing positions who had been involved in soccer training/play at least 2 days (d) per week (wk) for 6 months (mo) prior to the study. Each participant completed a physical activity readiness questionnaire (PAR-Q+) [25] and a health information form to minimize any contraindications to exercise. All potential risks were explained fully prior to any testing, and the participants provided written, informed consent of the study protocol which was approved by the Western University’s Office of Research Ethics and registered at clinical trials.gov, NCT04576026.

### 2.2. Preliminary Sessions

Participants visited the laboratory on two separate occasions prior to any experiments for familiarization sessions. During the initial visit, body composition (Bod Pod^®^, COSMED, Concord, CA, USA) $\dot{V}$O_2_ max (running treadmill test), and maximal sprinting speed (treadmill test) were determined. Briefly, $\dot{V}$O_2_ max was determined via an incremental speed protocol on a treadmill (Desmo Pro, Woodway^®^, Waukesha, WI, USA). Participants started running at 9.7 km·h^−1^ (6 mph) with the treadmill set at a constant grade of 1%. Subsequently, increases in speed of 0.16 km·h^−1^ (0.1 mph) every 12 s (s) were applied until volitional fatigue. Heart rate (HR) was monitored throughout the test (Polar RST200^TM^, Polar Electro Inc., Lachine, QC, Canada) and expired gases were analyzed via a breath-by-breath collection system (Sensormedics Vmax 29, Yorba Linda, CA, USA), calibrated according to manufacturer’s guidelines using known gases volumes and composition. The greatest value achieved over a 20 s collection period was considered max whenever a plateau in $\dot{V}$O_2_ occurred (<50% of the expected increase in oxygen uptake for the increased workload) or when two of the following three criterion measures were attained (±10 bpm of age predicted maximum HR, RER > 1.15 [RER = volume of CO_2_ produced/volume of O_2_ consumed] or volitional fatigue). Further, peak sprint speed was determined using a 10–15 s all-out effort on a non-motorized treadmill (Desmo Pro, Woodway^®^, Waukesha, WI, USA). Briefly, at a 2% incline with a disengaged belt, each participant propelled the belt themselves and the greatest speed shown on the treadmill display board during the test was recorded as peak speed.

At least 48 h following the first visit, participants returned to the lab a second time to familiarize themselves with both the cognitive tests (Stroop and Choice Reaction Test (CRT)—details below) and the high intensity, intermittent running protocol used to simulate the second half of the soccer match. This was done to eliminate potential learning effects during the experimental sessions.

### 2.3. Experimental Sessions

**Figure 1:**
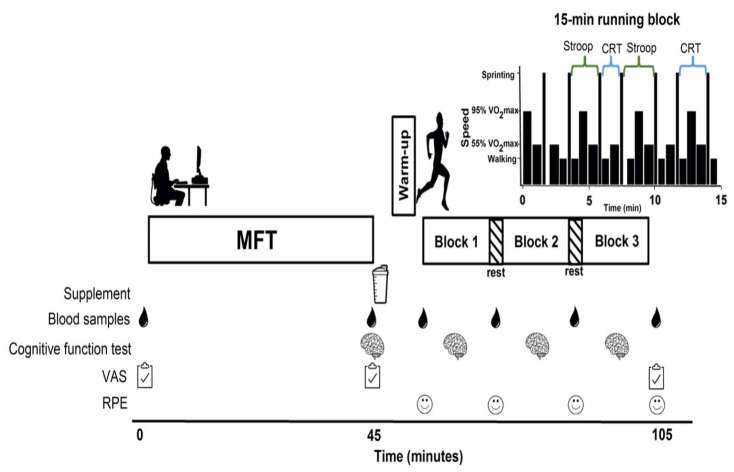
Overview of study protocol. MFT = mental fatiguing task. Cognitive Function Test included the Stroop and Choice Reaction Test.

A double blind, treatment order balanced, crossover research design was implemented involving two experimental sessions that comprised two treatments, KME (25 g [329 ± 34 mg·kg^−1^ with a range of 297 mg·kg^−1^–398 mg·kg^−1^] ΔG ketone performance, TdeltaS Global, Orlando FL, USA) and a non-caloric appearance-matched placebo (PLAC) (Crystal Light, Kraft, Toronto, ON, Canada). Supplements were prepared by an individual not otherwise involved in the study. The treatment blinding was successful (only 3 out of 9 participants guessed the treatment correctly). Experimental sessions were comprised of a 40-min mental fatiguing task (MFT), followed by a 45-min SSM. Cognitive function, blood metabolites, rates of perceived exertion, mental fatigue and mental effort were measured before, during and after exercise (Figure 1). The two experimental sessions were separated by at least one wk, were conducted at the same time of the d, and were rotated systematically to prevent order effects. Further, to minimize food intake differences across treatments, participants recorded their entire food/drink intake for the d preceding and the d of the first experimental session and replicated this intake for their second trial.

Participants reported to the laboratory 4 h postprandial with limited activity (drive/use of the elevator to get to the laboratory), having abstained from strenuous exercise, caffeine, or alcohol consumption for 24 h. This dietary control was utilized not only to standardize conditions between treatments but also to ensure that participants were able to exercise in a fed state, as this is how athletes engage in competition typically. Upon arrival, capillary blood samples for baseline glucose, beta-hydroxybutyrate (βHB) (FreeStyle Precision Neo^®^, Abbott Diabetes Care Limited, Mississauga, ON Canada) and lactate (Lactate Scout+, EKF Diagnostics, Cardiff, UK) concentration were obtained using the fingerstick method. Subsequently, baseline ratings of mental fatigue were collected using a 100 mm visual analog scale (VAS) and then, the MFT commenced. For this task, a longer version of the Stroop test (40-min) was implemented as this approach has been shown to induce a state of mental fatigue previously [7]. Following the MFT, pre-exercise cognitive function was assessed using both the Stroop and CRT. Furthermore, ratings of mental fatigue and mental effort were collected using a 100 mm VAS, as described by Smith et al. [7]. Next to assess blood metabolites, capillary blood samples were collected and the corresponding treatment (KME or PLAC) was given. Both drinks were 125 mL in volume and were provided in plastic cups. Five min after drink ingestion, a 5-min warm-up was completed, followed by a 45-min SSM (Figure 1). The SSM was comprised of 3 × 15-min intermittent running blocks interspersed with 3-min of passive recovery. The intensities and times used during each block were full stopping (15 s), walking (35 s), jogging (46 s at 55% $\dot{V}$O_2_ max), cruising (42 s at 95% $\dot{V}$O_2_ max) and sprinting (17 s at 90% of peak sprint speed). We have used this protocol previously [8,26] and it has been shown to replicate the physiological demands of soccer play [27]. HR was monitored throughout using a Polar RST200^TM^ (Polar Electro Inc., Lachine, QC, Canada). Capillary blood samples to measure glucose, βHB and lactate, as well as ratings of perceived exertion (RPE—6–20 Borg scale) were taken before the simulated match and during each 3-min passive recovery throughout. After providing the supplements, only one investigator remained in the room with the participants to run the equipment and to ensure testing happened in a distraction-free environment. The laboratory temperature was 21–22 °C for all experiments.

### 2.4. Cognitive Function

Two cognitive function tests were used, the Stroop and CRT. The Stroop test was used for the MFT (40-min) to induce mental fatigue, and together with the CRT during the exercise bout to assess cognition. A computer screen was set up in front of the treadmill and a control box (four buttons to respond to the stimuli presented on the computer screen) was placed in front of the participant.

#### 2.4.1. The Stroop Test

Briefly, words of four colours (red, blue, yellow, and green) were displayed in the center of the computer screen written in either congruent (i.e., word ‘blue’ written in blue ink) or incongruent ink (i.e., word ‘blue’ written in some other colour ink). Participants responded by pushing the button that matched the color displayed on the computer screen, not the word. This test was performed twice within each exercise block for a total of 240 trials (120 congruent and 120 incongruent) during the SSM. The Stroop effect was quantified as the difference in mean choice-reaction time between congruent and incongruent trials. The task was scored as the difference in mean choice reaction time (ms), accuracy (%) and Stroop effect (ms) from baseline (mental fatigued state) relative to exercise.

#### 2.4.2. Choice Reaction Test (CRT)

Briefly, the computer screen was divided into four equal-area quadrants and each area was assigned a specific response key on the 4-button box located in front of the participants. Stimuli sporadically flashed on the screen and participants were instructed to respond as quickly as possible by pushing the button on the box corresponding to the location of the stimuli on the screen. This test was performed twice within each exercise block for a total of 480 trials during the SSM. The task was scored as the difference in mean choice reaction time (ms) and accuracy (%) from baseline (mental fatigued state) relative to exercise.

### 2.5. Statistical Analysis

Statistical analyses were performed using SigmaPlot for Windows (Version 12.5, SYSTAT, San Jose, CA, USA). Blood metabolite concentrations as well as ratings of perceived exertion, and mental fatigue were analyzed using two-way (condition by time) repeated-measures ANOVA. Post hoc Tukey’s Honest Significant Difference (HSD) testing was used, where necessary. Further, because we observed a treatment difference in some cognitive measures following the mental fatigue task vs. baseline but not at baseline and because our focus was on whether or not there was a treatment effect during exercise as opposed to the mental fatigue test, the differences between the post mental fatigue task and exercise values for mental effort, Stroop test, and CRT were analyzed using paired *t* tests. To do so, the exercise data were averaged. Significance was set at *p* ≤ 0.05. Data are presented as means ± SD.

## 3. Results

### 3.1. Blood Metabolites

**Figure 2:**
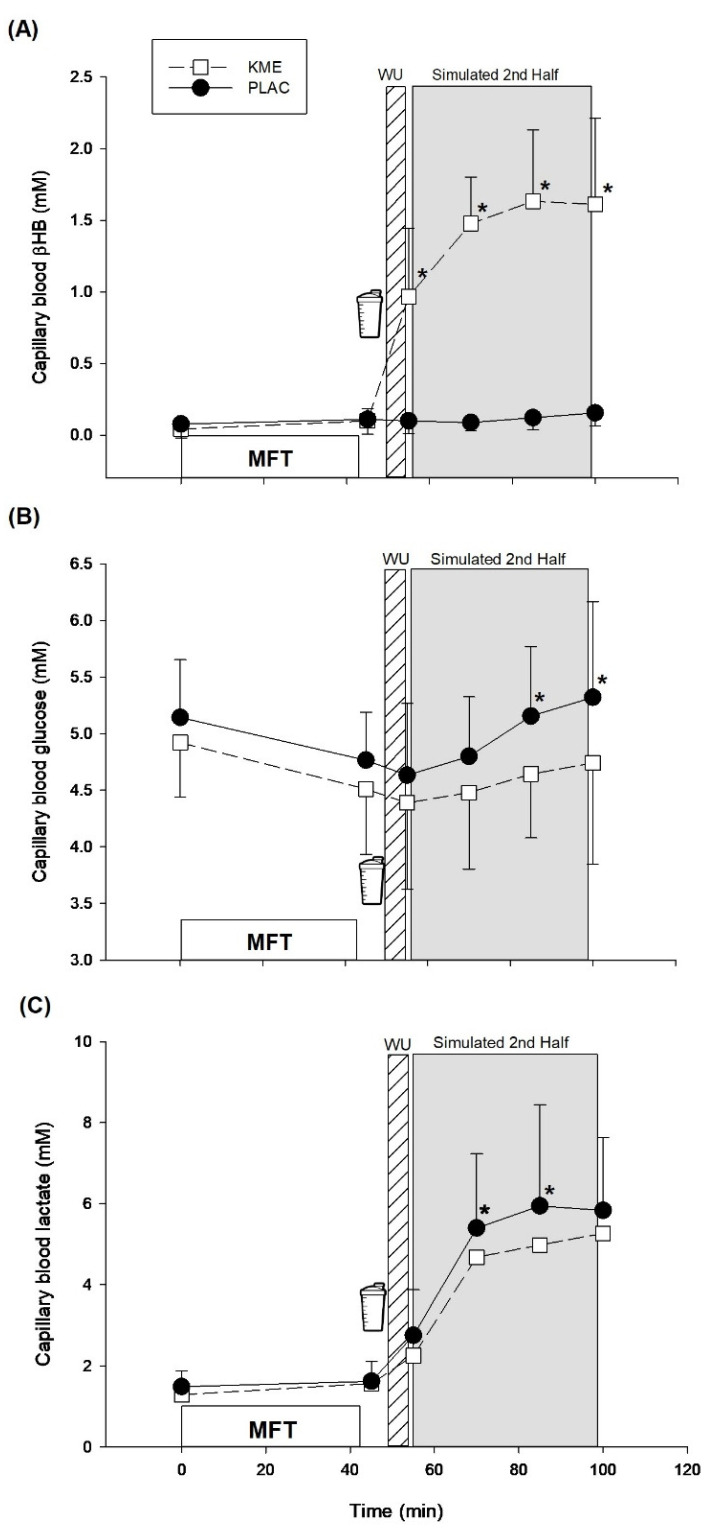
Effects of KME ingestion on blood metabolites during exercise after induced mental fatigue. (**A**) blood βHB concentration (**B**) blood glucose concentration (**C**) blood lactate concentration. Values are means ± SD. MFT = mental fatiguing task; WU = warm-up; KME = ketone monoester supplement; PLAC = non-caloric placebo. * Significantly different versus PLAC.

There was a significant treatment × time interaction (*p* < 0.001) for blood βHB (Figure 2A). Pairwise comparisons showed that blood βHB concentration was greater for KME after warm-up (*p* < 0.01) and during all 3 exercise blocks (*p* < 0.001), compared with PLAC. In addition, there were main effects of both time (*p* < 0.001) and treatment (*p* < 0.001) for blood βHB concentration. As expected, blood βHB was greater at every time point after supplement ingestion, compared to both baseline and post-MFT.

There was a significant treatment × time interaction (*p* = 0.02) for blood glucose (Figure 2B). Pairwise comparisons showed that blood glucose was greater for PLAC in block 2 (*p* = 0.02) and block 3 (*p* = 0.01), compared with KME. In addition, no main effect of treatment was found (*p* = 0.58) but there was a main effect of time (*p* = 0.02) for blood glucose concentration showing that blood glucose was lower post-MFT, compared to baseline.

A main effect of time (*p* < 0.001) was also detected for blood lactate (Figure 2C). As expected, lactate concentrations were greater during exercise relative to baseline and post-MFT. No treatment effect (*p* = 0.47) was observed but there was a treatment x time interaction (*p* = 0.02). Pairwise post hoc comparisons indicated that blood lactate concentration was greater for PLAC for exercise block 1 (*p* = 0.05), and block 2 (*p* = 0.01), compared with KME.

### 3.2. Visual Analog Scales

**Figure 3:**
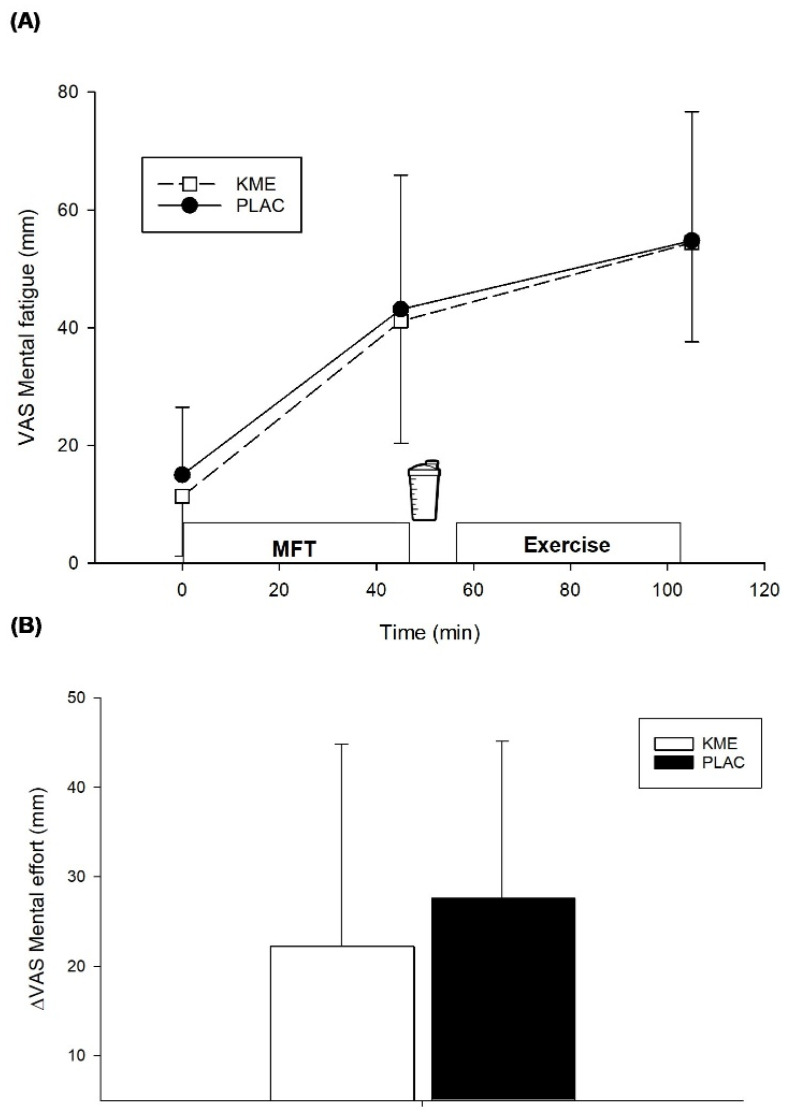
Effects of KME supplementation on subjective ratings of mental fatigue and mental effort (**A**) Subjective ratings of mental fatigue (**B**) difference in subjective ratings of mental effort, relative to pre-exercise (Post MFT). Values are means ± SD. MFT = mental fatiguing task; KME = ketone monoester supplement; PLAC = non-caloric placebo. VAS = Visual Analogue Scale.

Subjective ratings of mental fatigue increased significantly in both trials after the mental fatiguing task (*p* = 0.003) and after exercise (*p* < 0.001), compared to baseline. However, no differences were found between KME and PLAC at any time point (*p* = 0.53) (Figure 3A).

Subjective ratings of mental effort were similar under both conditions (*p* = 0.47) (Figure 3B).

### 3.3. Ratings of Perceived Exertion (RPE)

A main effect of time (*p* < 0.001) was detected for RPE (Table 1). Not surprisingly, average RPE for block 2 and block 3 were greater than both pre-exercise and block 1. No treatment effect (*p* = 0.90) nor treatment x time interactions were observed (*p* = 0.13).

### 3.4. Cognitive Function

Stroop Test: There were no differences in reaction time for congruent (*p* = 0.78) or incongruent (*p* = 0.43) trials, as well as no differences in the percent of correct answers for congruent (*p* = 0.38) and incongruent (*p* = 0.16) trials. Lastly, there were no differences in Stroop effect (*p* = 0.44) when comparing KME to PLAC group (Table 2).

**Figure 4:**
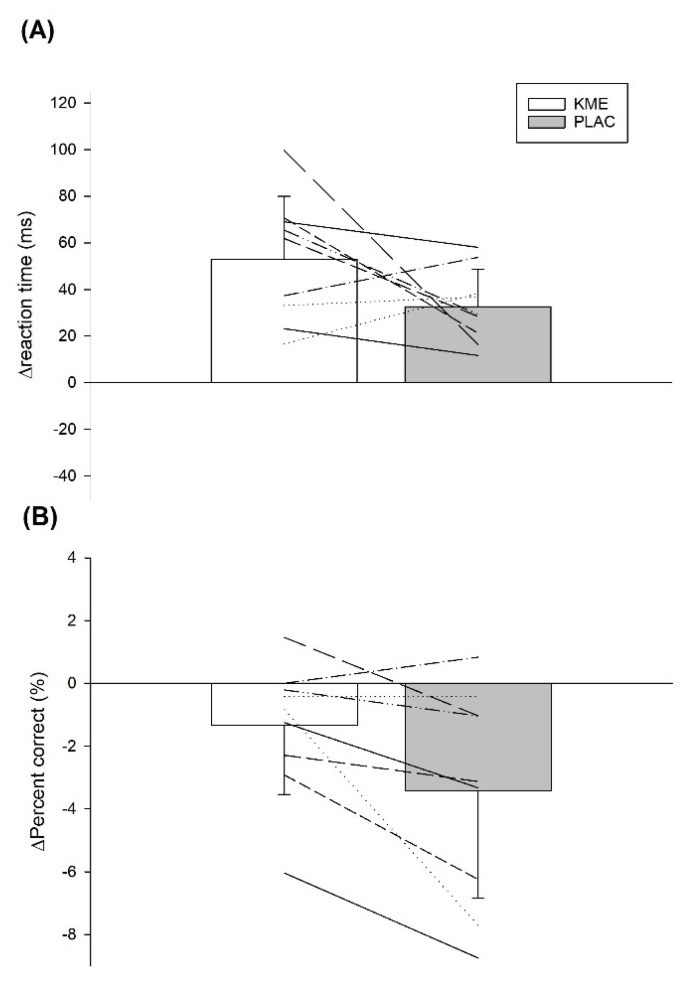
Effects of KME supplementation on cognitive function (CRT) during exercise after induced mental fatigue, compared to PLAC. (**A**) reaction time during exercise, relative to pre-exercise (Post MFT) (**B**) %correct answers during exercise, relative to pre-exercise (Post MFT). Values are means ± SD. Thin lines indicate individual participant data. MFT = mental fatiguing task; KME = ketone monoester supplement; PLAC = non-caloric placebo.

Choice Reaction Test: Compared to PLAC, the KME group displayed a reduced decrease in the percent of correct answers during exercise (*p* = 0.02), while maintaining a similar increase in reaction time (*p* = 0.11), indicating a better maintenance of cognitive function with KME (Figure 4).

## 4. Discussion

The purpose of the present study was to assess the efficacy of acute KME supplementation on cognitive performance during a 45-min SSM after induced mental fatigue. Interestingly, our results revealed that relative to pre-exercise, the number of correct answers was better maintained with KME during exercise compared to PLAC, while reaction time remained similar in the CRT. In contrast, no differences were seen with the Stroop test when comparing both treatments. We analyzed change scores (post mental fatigue task vs. exercise) for these cognitive measures because some differences were seen in the number of correct answers for the CRT following the mental fatigue task (KME: 95% correct vs. PLAC: 98%), although there were no differences at baseline. We did so because our main interest was in assessing any changes in cognitive function post mental fatigue vs. exercise. KME supplementation also elevated blood βHB, lowered blood glucose during the last 30-min of exercise, and resulted in reduced blood lactate during the first 30-min of exercise, perhaps indicating a shift in exercise fuel use away from CHO.

Previous investigations evaluating the effects of ketone supplements on cognitive function during exercise have produced mixed results, with a few studies showing positive outcomes [18,19], and others showing no benefits [20,21,22]. As mentioned, part of the discrepancy has been attributed to factors such as the type of supplement used (KS vs. KE) or differing methodology [23]. For example, KE have been shown to induce a greater ketonemia compared to KS [16] and this is likely important because the magnitude of ketosis (in the range of ∼1 to 3 mM) has been deemed essential to induce certain physiological changes [11]. In the present study, participants ingested KME and reached a blood βHB concentration of ~1.6 mM. A previous study that also found cognitive function improvements provided KME at a even greater dose (750 mg·kg^−1^) and generated blood βHB concentrations of ~2.6 mM [18]. In contrast, Walmand et al. [20,22] provided KS and reported no benefit on cognitive function. In these studies, participants achieved a blood βHB concentration of ~0.5 mM [20] and ~0.8 mM [22]. Furthermore, the difference in the mode of exercise (intermittent vs. continuous) as well as the time when the cognitive tasks are administered may also affect the outcomes. For instance, Evans and Egan [18] demonstrated that supplementing with KME and CHO before and during a ~90-min intermittent exercise session reduced the number of incorrect responses during a cognitive test administered after exercise, when compared to CHO alone. However, the same authors found no benefit when these cognitive tasks were performed after a 10 km time trial [21]. Perhaps exercise mode and duration contributed to these differing results. In the present study, participants performed only 45-min of intermittent exercise, although it was done in a state of pre-induced mental fatigue. Our findings are consistent with Evans and Egan [18], since participants ingesting KME in our study were able to reduce the number of incorrect answers relative to baseline during the CRT, compared to PLAC. This is likely important for many sporting activities because CRT has been shown to provide insight on individual decision-making under pressure [28]. Further, Evans and Egan [18] administered their cognitive task before and after exercise, while in the present study it was done concurrently with exercise, something that enhances ecological validity, and this timing difference may be important. For example, Lambourne and Tomporowski [24] observed small improvements in cognitive function when tests were completed after exercise cessation whereas a slight impairment was seen when tests were done during exercise, something that would make any cognitive task more sensitive to detect a potential difference. However, this apparent benefit of KME supplementation was not detected with the Stroop test in the present study. Perhaps participants accrued some learning during the mental fatiguing task which utilized the Stroop test, making the treatment test less sensitive to detect any differences. Consistent with Smith et al. [7] this approach was effective at increasing subjective ratings of mental fatigue (Figure 3A) but given the duration of the task, it could be that our participants became accustomed to the stimuli presented, making it less likely to detect an effect during exercise, if any. Nonetheless, these results should be interpreted with caution and additional study with more challenging dynamic visual acuity tasks [29] or even with future sport video game simulation tasks using the technology now available from companies like EA Sports (https://www.ea.com/ea-studios/ea-sports (accessed on 25 September 2022)) is needed to confirm the present findings

Interestingly, metabolic responses appeared to differ in our study between the two treatments. As expected, KME ingestion elevated blood βHB during exercise vs. PLAC, an effect that has been consistent across studies using KME supplementation [12,23]. Furthermore, KME lowered blood glucose in the last 2 blocks of exercise, and reduced blood lactate during the first 2 blocks of exercise, These metabolic responses are consistent with the majority of studies using KME supplementation during exercise [15,18,30]. In particular, one study (18) provided KME and found small improvements in cognitive function. In contrast, another study examining cognitive function with KME did not observe similar metabolic responses nor any differences in cognition [21]. Some have argued that these metabolic responses are indicative of a potential CHO sparing effect, largely because the first published study found improvements in physical performance following KME ingestion [15]. However, subsequently it has been suggested that high blood βHB concentrations would be expected to lower pH and these perturbations to acid-base homeostasis could reduce the rate of glycolysis and thus lactate production [30]. Of course, this could affect soccer performance adversely. However, it is important to note that these perturbations may occur only when blood βHB concentration is raised substantially. For example, Dearlove et al. [30] found that power output did not differ at high exercise intensities with a blood βHB concentration of ~3.7 mM. In our study, average blood βHB concentration was ~1.6 mM during exercise, so it is plausible that any acidosis-induced detriments would be minimal. If so, the present data may be due to a CHO sparing effect rather than an inhibition of glycolysis. A recent study showed that compared to an isoenergetic CHO supplement, coingestion of KME and CHO resulted in a 2.1% improvement in high-intensity intermittent performance, while no difference in sprint performance was observed during a rugby simulation protocol [31]. Unfortunately, physical performance was not assessed directly in the present study so whether the aforementioned differences might enhance exercise performance must await further experimentation.

Although the present study had a sufficient sample size (n = 9) based on sample size calculations using data from Smith et al. [7], it was still small. Further, our protocol was designed to simulate the soccer match environment as much as possible, for example, by administering cognitive tests and running concurrently, by having participants exercise in a fed state, etc. However, the laboratory setting, motorized treadmill and, most importantly the lack of soccer-specific play, limits the ecological validity of our study. Further, our cognitive function results showed differences in CRT only and not in the Stroop test, perhaps indicating that KME improved only one cognitive domain. Finally, our treatments were not matched for taste nor energy. The purpose with this experiment was to isolate the potential positive effects of KME supplementation on cognitive function during exercise. Thus, a comparison was made vs. a non-caloric, flavoured placebo. Although taste differences were likely unimportant as only 33% of our participants correctly identified the treatment, it is possible that energy differences influenced our results.

In summary, acute KME supplementation elevated circulating βHB during intense repeated, intermittent exercise and produced lower exercise concentrations of both glucose and lactate vs. a non-caloric placebo. Moreover, KME supplementation resulted in fewer cognitive errors after induced mental fatigue relative to baseline in the CRT, while no differences were observed in any other cognitive variable. These data suggest that KME supplementation can attenuate the decline in some aspects of cognitive function during sports characterized by repeated, intense, intermittent exercise. More study is needed to assess fully the possible cognitive/physical benefits of KME for athletes.

## Figures and Tables

**Table 1:** Ratings of perceived exertion (RPE).

	KME	PLAC	Two-Way RM ANOVA	
Pre-SSM	9.3 ± 1.0	9.9 ± 1.1	Time Effect	*p* < 0.001
Block 1	13.2 ± 1.0 ^a^	12.8 ± 1.1 ^a^	Treatment Effect	*p* = 0.90
Block 2	14.7 ± 1.4 ^b^	14.8 ± 1.4 ^b^	Interaction Effect	*p* = 0.13
Block 3	15.9 ± 1.8 ^b^	15.8 ± 1.6 ^b^		

Values are means ± SD. RPE data were analyzed using two-way repeated measures ANOVA. Post hoc Tukey’s HSD testing was used to assess differences between treatments. There were no significant differences between KME and PLAC (*p* = 0.13). SSM = Simulated Soccer Match. ^a^ indicates significant increase from Pre-SSM. ^b^ indicates significant increase from Pre-SSM and Block 1.

**Table 2:** Stroop test.

Variable Measured		Treatment	∆Score	*p*-Value
	RT (ms)	KME	26.7 ± 60.4	0.78
Congruent		PLAC	22.4 ± 50.2	
	Percent Correct (%)	KME	3.6 ± 4.7	0.38
		PLAC	1.9 ± 2.2	
	RT (ms)	KME	24.9 ± 111.4	0.43
Incongruent		PLAC	0.31 ± 62.0	
	Percent Correct (%)	KME	4.4 ± 5.4	0.16
		PLAC	5.4 ± 5.6	
	Stroop effect	KME	−1.8 ± 68.3	0.44
		PLAC	−22.1 ± 71.6	

Values are means ± SD. The difference in pre-exercise Stroop task (post MFT) and during exercise were calculated for all variables (∆Score). Data were analyzed using a paired *t* test. There were no significant differences between KME and PLAC.

## Data Availability

The authors will make deidentified raw data set available upon reasonable requests.
